# Bioinformatic Study of Possible Acute Regulation of Acid Secretion in the Stomach

**DOI:** 10.1007/s00232-024-00310-7

**Published:** 2024-03-04

**Authors:** Yan Hay Grace Lee, Nicole T. Cerf, Nicholas Shalaby, Mónica R. Montes, Ronald J. Clarke

**Affiliations:** 1 School of Chemistry, University of Sydney, Sydney, NSW 2006 Australia; 2 Instituto de Química y Fisicoquímica Biológica (IQUIFIB), CONICET, Universidad de Buenos Aires, Buenos Aires, Argentina; 3 The University of Sydney Nano Institute, Sydney, NSW 2006 Australia

**Keywords:** Gastric proton pump, Mirror tree analysis, Amino acid sequence analysis, Protein kinase C, Src kinase, Electrostatic switch mechanism

## Abstract

The gastric H^+^,K^+^-ATPase is an integral membrane protein which derives energy from the hydrolysis of ATP to transport H^+^ ions from the parietal cells of the gastric mucosa into the stomach in exchange for K^+^ ions. It is responsible for the acidic environment of the stomach, which is essential for digestion. Acid secretion is regulated by the recruitment of the H^+^,K^+^-ATPase from intracellular stores into the plasma membrane on the ingestion of food. The similar amino acid sequences of the lysine-rich N-termini α-subunits of the H^+^,K^+^- and Na^+^,K^+^-ATPases, suggests similar acute regulation mechanisms, specifically, an electrostatic switch mechanism involving an interaction of the N-terminal tail with the surface of the surrounding membrane and a modulation of the interaction via regulatory phosphorylation by protein kinases. From a consideration of sequence alignment of the H^+^,K^+^-ATPase and an analysis of its coevolution with protein kinase C and kinases of the Src family, the evidence points towards a phosphorylation of tyrosine-7 of the N-terminus by either Lck or Yes in all vertebrates except cartilaginous fish. The results obtained will guide and focus future experimental research.

## Graphical abstract

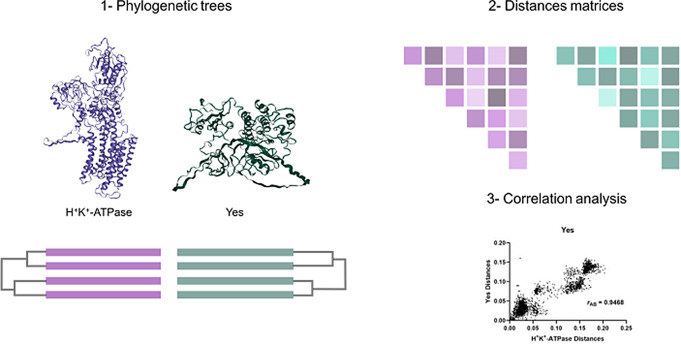

## Introduction

A low pH within the stomach is essential for the activation of pepsin, a stomach enzyme which breaks down the protein of ingested food. Acid secretion into the stomach is accomplished by the H^+^,K^+^-ATPase (or gastric proton pump), which is located in the plasma membrane of parietal cells of the gastric mucosa. In an electroneutral exchange of K^+^ ions from the stomach lumen, the H^+^,K^+^-ATPase utilises the energy of ATP hydrolysis to pump H^+^ ions into the stomach (Sachs et al. 1976). Thus, the H^+^,K^+^-ATPase plays a crucial role in the digestion process. However, the acidic environment of the stomach provided by the H^+^,K^+^-ATPase causes the bacterium *Helicobacter pylori*, which promotes the formation of peptic ulcers and even stomach cancer, to adopt a coccoid form resistant to antibiotic treatment (Ierardi et al. 2019). The discovery of the mechanism of H^+^ secretion (Sachs et al. 1976; Forte et al. 1975; Lee and Forte 1978; Wallmark and Mårdh 1979) and the subsequent development of inhibitors (proton pump inhibitors or PPIs), such as omeprazole, can be considered as a revolution in twentieth century medicine, with many millions of patients afflicted with peptic ulcers or gastroesophageal reflux disease being successfully treated (Sachs et al. 2014; Kaunitz 2014).

The gastric H^+^,K^+^-ATPase is a member of the P-type ATPase family of enzymes; a large group of integral membrane proteins found in all kingdoms of life which pump small ions, lipids and some other molecules across the membrane in which they are located. They derive the energy they require for the pumping process from the hydrolysis of ATP. They are, therefore, classified as primary active transporters. Transport stoichiometries of the H^+^,K^+^-ATPase of 2 H^+^/2K^+^ and 1 H^+^/1K^+^ per hydrolysed ATP have been reported, but with the stoichiometry accepted to be 1 H^+^/1K^+^ at low pH values of the stomach lumen (Reenstra and Forte 1981; Rabon et al. 1982, 1993; Abe et al. 2012; Montes et al. 2011; Yamamoto et al. 2019). The H^+^,K^+^-ATPase belongs to the P2C group of the P2 subfamily of P-type ATPases, as does the Na^+^,K^+^-ATPase. Both enzymes comprise a catalytic α-subunit and a much smaller β-subunit; in the case of the Na^+^,K^+^-ATPase there is a third even smaller subunit with a single membrane-spanning α-helix, which is termed the γ-subunit in kidney cells. The α-subunits of the H^+^,K^+^-ATPase and the Na^+^,K^+^-ATPase have a high degree of structural homology of approximately 63% (Shin et al. 2009). Therefore, one would expect similarities in the function and regulation of the two enzymes, and hence comparisons in their behaviours to yield useful information.

The primary mode of regulation of acid secretion in the stomach involves recruitment of the H^+^,K^+^-ATPase from intracellular tubovesicular elements to the apical membrane surface of stimulated parietal cells upon the ingestion of food (Dunbar and Caplan 2001). Once at the apical membrane surface, the H^+^,K^+^-ATPase fuses with the plasma membrane so that it can start pumping H^+^ ions into the stomach lumen. This poses the question, is there any acute regulation of acid secretion via direct modulation of H^+^,K^+^-ATPase transport kinetics? In vitro biochemical studies on the H^+^,K^+^-ATPase and comparisons with the Na^+^,K^+^-ATPase suggest that this may be the case (Cornelius and Mahmmoud 2003).

Common to both catalytic α-subunits of the H^+^,K^+^-ATPase and the Na^+^,K^+^-ATPase is a lysine-rich cytoplasmic N-terminal tail. In the case of the Na^+^,K^+^-ATPase this tail possesses a tyrosine and a serine residue which are conserved across all vertebrates (Diaz and Clarke 2018). These are potential sites for acute regulation of ion pumping activity via phosphorylation by an Src kinase and a protein kinase C (PKC), respectively (Blayney et al. 2023). Tyr-5 (-10 if the propeptide sequence is included in the numbering) has been identified in cultured kidney cell studies as a Src kinase target (Petrič et al. 2021). Similarly, Ser-11 (-16 if the propeptide sequence is included) has been identified in studies on both purified Na^+^,K^+^-ATPase from kidney (Feschenko and Sweadner 1995) and on oocyte homogenates expressing the Na^+^,K^+^-ATPase (Beguin et al. 1994) to be a target of PKC. Based on both experimental data and theoretical molecular dynamics simulations, it has been proposed that the regulation occurs via an electrostatic switch mechanism. This involves salt bridge-type interactions between N-terminal positively charged lysine residues and negatively charged lipid headgroups, such as phosphatidylserine, that are weakened by the kinase-induced introduction of the negatively charged phosphate moiety onto the conserved tyrosine and serine residues (Blayney et al. 2023; Lev et al. 2023). Weakening of the salt bridge interactions with the membrane surface is proposed to change the relative stabilities of different conformational states of the Na^+^,K^+^-ATPase α-subunit and thus lead to a change in ion pumping activity.

The α_1_-subunit N-terminus of the H^+^,K^+^-ATPase also possesses tyrosine and serine residues in many vertebrate species, but they are not as broadly conserved as in the Na^+^,K^+^-ATPase (Diaz and Clarke 2018). Ser-27 (*Homo sapiens* sequence numbering) appears to be conserved in all placental mammals. Tyr-7 seems to be conserved in all vertebrates except cartilaginous fish, and a tyrosine is also present in position 10 in most mammals (including marsupials), but it is replaced by histidine in some species. Similar to the Na^+^,K^+^-ATPase, in vitro experiments (Togawa et al. 1995, 1996; Kanagawa et al. 2000; Fujitani et al. 2003) suggest that some of these residues could be sites of regulatory phosphorylation by kinases in many species. Asano et al. (2000), however, carried out experiments with the H^+^,K^+^-ATPase expressed in HEK-293 cells, in which they replaced all of the positively charged lysine residues of the N-terminus with uncharged alanine residues. This had no effect on the stimulation of the enzyme’s steady-state ATPase activity by ATP, K^+^ or H^+^. However, these results don’t exclude a role of the N-terminus in determining ion pump kinetics, because there remains the possibility that the N-terminus could play a role in the conformational change of the unphosphorylated enzyme, which is necessary for K^+^ deocclusion and release into the cytoplasm. That the N-terminus could affect this transition would seem reasonable, considering its location on the cytoplasmic face of the protein. A significant role of the N-terminus of the H^+^,K^+^-ATPase α-subunit was in fact reported by Cornelius and Mahmmoud (2003), who found that N-terminal phosphorylation by PKC caused a 40–80% increase in ATPase activity at saturating ATP concentrations, depending on the pH. The number of kinetic studies on partial reactions of the H^+^,K^+^-ATPase is much less than that of the Na^+^,K^+^-ATPase, but stopped-flow measurements show that in the absence of ATP, the E2 → E1 conformational transition associated with K^+^ deocclusion to the cytoplasm is 50–100 times faster in the H^+^,K^+^-ATPase than in mammalian Na^+^,K^+^-ATPase (Rabon et al. 1990; Faraj et al. 2021). This does not exclude, however, a contribution of the E2 → E1 transition to rate determination of the H^+^,K^+^-ATPase ion pumping cycle under physiological conditions in the presence of ATP. It is interesting to note here that a higher rate of the E2 → E1 transition is also seen in Na^+^,K^+^-ATPase from shark (*Squalus acanthias*) rectal gland (Khalid et al. 2010). Another similarity between the shark Na^+^,K^+^-ATPase and the H^+^,K^+^-ATPase is that the α-subunit of both have longer cytoplasmic N-termini with even more lysine residues than that of mammalian Na^+^,K^+^-ATPase α_1_-subunits (Diaz and Clarke 2018). Therefore, it’s possible that the different kinetic behaviours observed with respect to the E2 → E1 conformational transition could be due to differences in the amino acid sequences of the N-termini of the enzymes. No three-dimensional structural data is available on the N-terminus of the H^+^,K^+^-ATPase because it was removed prior to crystallisation, probably to facilitate crystal formation due to the disordered nature of the N-terminus (Abe et al. 2018).

Although experimental data from in vitro experiments support the conclusion that the H^+^,K^+^-ATPase could undergo acute regulation via interaction of PKC and the Src kinase with the α-subunit N-terminus, in vivo evidence is lacking. A novel approach to tackling this problem has recently been used on the Na^+^,K^+^-ATPase (Blayney et al. 2023). The approach is based on the idea that if two proteins interact with one another, they should be co-evolving, such that if one protein undergoes a mutation, the other protein should undergo a compensating mutation to maintain optimal interaction. Thus, an analysis of the degree of coevolution of the H^+^,K^+^-ATPase with protein kinase C and with the Src kinase could potentially provide an indication of whether the enzymes interact with one another in vivo. The bioinformatic method that we use to analyse for co-evolution is called the mirror tree method, because it involves determining the similarity of the two proteins’ phylogenetic trees. Using this method it could be shown that, based on the analysis, the θ and η isoforms of PKC are the isoforms most likely to interact with the Na^+^,K^+^-ATPase, and that, of the kinases of the Src family, the Src kinase itself was the most likely reaction partner (Blayney et al. 2023). Thus, not only does the method allow one to analyse whether kinase regulation likely occurs in vivo, if it does, the method also allows the most likely kinase isoform involved to be identified. This is then very useful information that could be used to guide future experimental investigations.

Here we present mirror tree analyses of the coevolution of the α_1_-subunit of the H^+^,K^+^-ATPase with all 10 isoforms of PKC (α, β1, β2, γ, δ, ε, η, θ, ζ and λ(ι)) and all 9 members of the Src kinase family (Src, Yes, Fyn, Fgr, Lck, Hck, Blk, Lyn and Frk). We also present an expanded data set of amino acid sequence alignments of the H^+^,K^+^-ATPase α_1_-subunit to determine the degree of conservation of possible regulatory kinase targets. The results support the conclusion that the H^+^,K^+^-ATPase could undergo acute regulation by kinase phosphorylation in many animal species.

## Methods

### H^+^,K^+^-ATPase Amino Acid Sequence Analysis

Sequences of the main catalytic α subunit of the gastric H^+^,K^+^-ATPase were obtained from the protein database of the National Center for Biotechnology Information (https://www.ncbi.nlm.nih.gov/protein/) and from the UniProt database. All available entire vertebrate sequences were aligned using the MUSCLE program (Edgar 2004) within the MEGA11 suite of evolutionary genetics programmes (Kumar et al. 2016).

### Mirror Tree Analysis

The evolutionary relatedness of the H^+^,K^+^-ATPase and various isoforms of PKC and Src were investigated using a distinct computational approach, namely mirror tree analysis, which analyses the similarity of two proteins’ phylogenetic trees (Pazos and Valencia 2001; Ochoa and Pazos 2010; Ochoa et al. 2015).

Amino acid sequences of pairs of proteins of interest (e.g., the H^+^,K^+^-ATPase and different isoforms of protein kinase C (PKC), which phosphorylate serine (S) or threonine (T) residues) were obtained from the Uniprot protein database using a stringent BLAST search as described previously (Blayney et al. 2023). To determine whether PKC could interact with the N-terminus of the H^+^,K^+^-ATPase, each of the isoforms of PKC (α, β, γ, δ, ε, η, θ, ζ and λ (alternatively known as ι) were analysed against the catalytic α_1_-subunit (containing the N-terminus) of the H^+^,K^+^-ATPase. Two negative controls were used: (1) Hexokinase (a carbohydrate kinase not a protein kinase) and the H^+^,K^+^-ATPase, and (2) PKCα and epidermal growth factor receptor (EGFR), a protein that is not phosphorylated by serine/threonine kinases like PKC (Endres et al. 2014). For the positive control, the α_1_ and β_1_ subunits of the H^+^,K^+^-ATPase were used because these have a direct chemical interaction with one another.

For the two proteins of interest, the amino acid sequences collected were filtered for common species, typically returning approximately 100 common species for each pair of proteins to be analyzed. The filtered amino acid sequences were aligned using the MUSCLE program (Edgar 2004) within the MEGA-11 suite of evolutionary genetics programs (Kumar et al. 2016), and the evolutionary distances or pairwise distances were calculated. The pairwise distance represents the number of amino acid substitutions per site between two homologous proteins, i.e., the number of substitutions divided by the total number of amino acids in the sequence. In terms of a phylogenetic tree, the pairwise distance is defined as the sum of the lengths of each branch of the tree connecting two species. For each protein the complete set of pairwise distances was exported as a distance matrix. A correlation coefficient, r_AB_, between the pairwise distances of each protein was calculated via the Pearson method, as described previously (Blayney et al. 2023). The number of pairings of the common organisms (or the number of elements in a matrix of the common organisms), n, is related to the number of common organisms, N, by n = (N^2^ – N)/2. The closer the value of r_AB_ is to 1, the higher the likelihood that the two proteins co-evolved. More precisely, if there is a high correlation, it means that there is a tight relationship between changes in evolutionary rates for the two families of proteins along different branches of their phylogenetic trees. For example, if one protein experiences a rate increase along one branch, then the other protein also experiences a rate increase along the same branch. Shared patterns of rate variation suggest that the proteins have evolved in a correlated manner, perhaps due to similar adaptive pressures.

To compare correlation coefficients obtained for different pairs of proteins we first converted the r_AB_ values using Fisher’s r-to-z transformation to obtain a normal distribution, as described previously (Blayney et al. 2023). The two-tailed probability of the null hypothesis, P, that two correlation coefficients are not significantly different was then calculated from the corresponding z values and the associated number of pairs of organisms, N, using the on-line Vassarstats Website for Statistical Computation (http://vassarstats.net/) via the tool “Significance of the Difference between Two Correlation Coefficients”. Analysis of individual correlation coefficients were also carried out using the Vassarstats Website via the tool “Significance of a Correlation Coefficient”. In this case P represents the probability of the non-directional null hypothesis that there is no significant correlation between the evolutionary distances of the two proteins being compared.

## Results

### H^+^,K^+^-ATPase Amino Acid Sequence Analysis

**Fig. 1:**
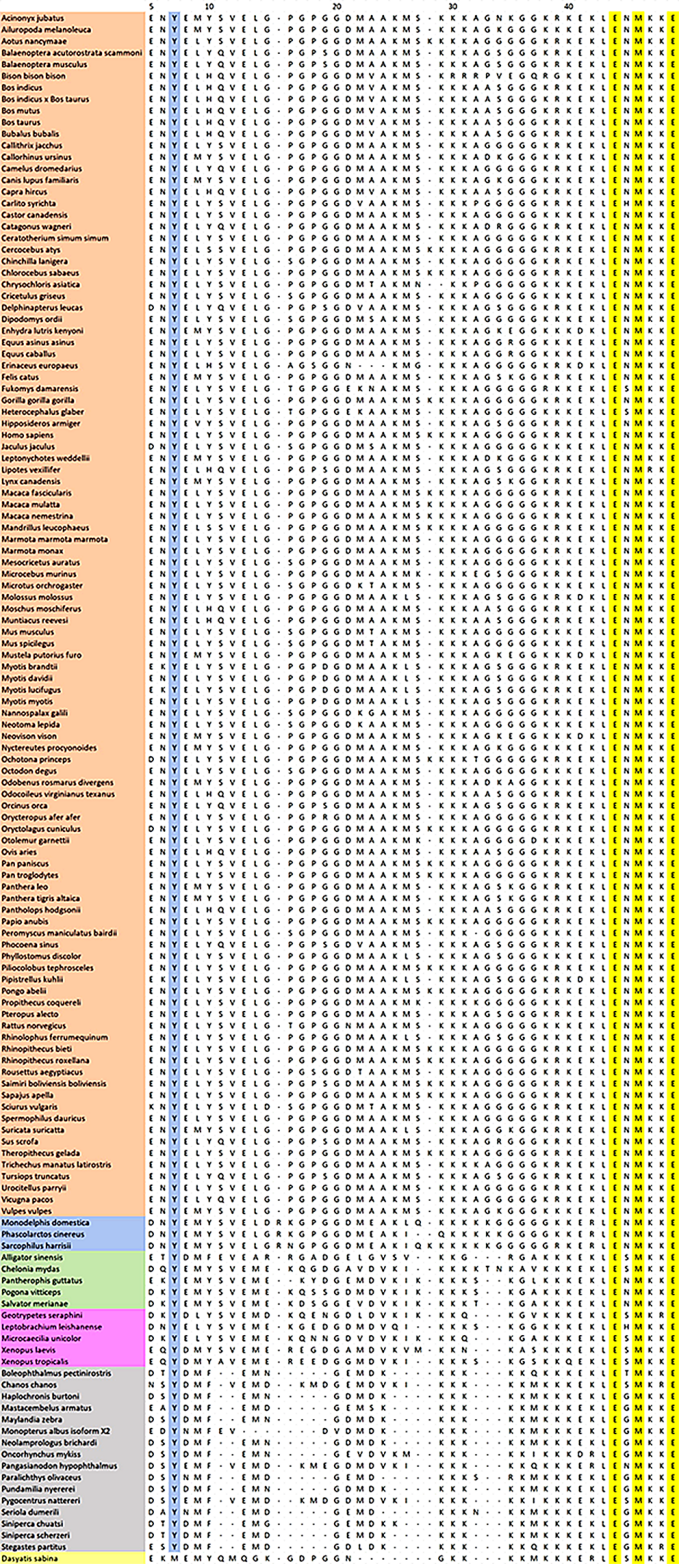
Sequence alignment of the N-terminus of the α_1_ isoform of the catalytic α-subunit of the gastric H^+^,K^+^-ATPase from vertebrates. Residues which are conserved across all species are highlighted in yellow. Tyrosine 7 (Y), which is conserved in all vertebrates except cartilaginous fish, is highlighted in light blue.The numbering of the residues is based on the *H. sapiens* sequence. The species have been grouped according to classes. i.e., placental mammals (orange, top), marsupial mammals (blue), reptiles (green), amphibians (pink), bony fish (grey) and cartilaginous fish (pale yellow, bottom)

To determine how widely distributed the amino acid targets of regulatory kinases are, here we have expanded the sequence database used previously (Diaz and Clarke 2018). The three amino acid residues which have been proposed (Togawa et al. 1995, 1996; Kanagawa et al. 2000; Fujitani et al. 2003) as kinase targets are Tyr-7, Tyr-10 and Ser-27. Of these residues, the most widely represented (see Fig. 1) is Tyr-7 (Y) which is represented in all species for which sequences were collected except for that of the cartilaginous fish, *Dasyatis sabina*. This would support the hypothesis that Tyr-7 could potentially be involved in direct regulation of H^+^,K^+^-ATPase by a tyrosine kinase, but that either the regulatory mechanism evolved after that of bony fish or that the mechanism was lost at some later stage by cartilaginous fish.

In contrast, Tyr-10, although present in many species, particularly mammals, is not a conserved residue. In many mammalian species, a histidine (H) occurs in this position and in all bony fish it is phenylalanine (F). This would argue against the involvement of this residue in a broadly represented regulatory mechanism.

The situation is similar with Ser-27. Previously it was thought that this residue was present in this position in all placental mammals (Diaz and Clarke 2018). But, assuming that there are no errors in the sequencing, in the expanded database that we’ve collected here, this residue is substituted in other placental mammals by asparagine (N) in *Chrysochloris asiatica*, by glycine (G) in *Erinaceus europaeus*, and by lysine (K) in *Microcebus murinus*, *Ottolemur garnetii* and *Propitheus coquereli*. It would, therefore, seem to be unlikely that Ser-27 is involved in a broadly represented direct regulation of the H^+^,K^+^-ATPase amongst placental mammals.

### Mirror Tree Analysis Controls

**Fig. 2:**
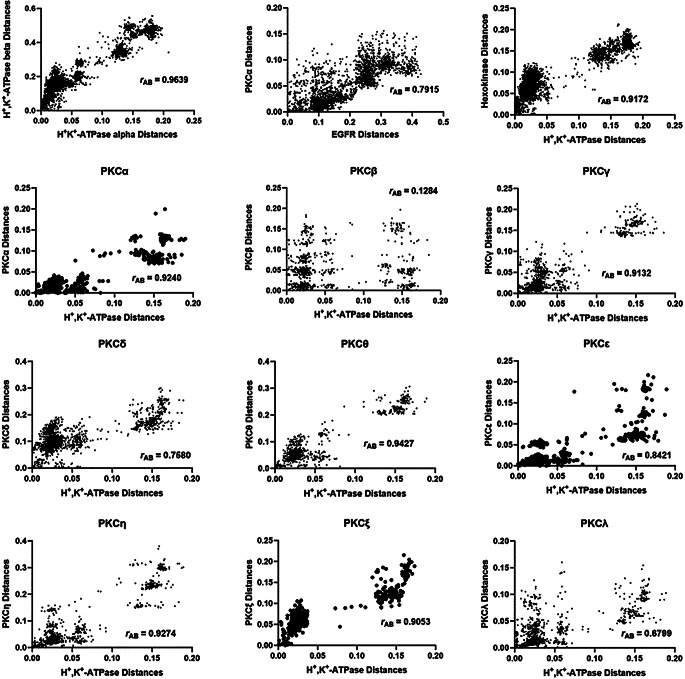
Correlations of evolutionary distances between common organisms expressing various isoforms of PKC and the H^+^,K^+^-ATPase α_1_-subunit. Each data point represents a combination of a pair of common organisms for each protein, as explained in the Materials and Methods section. Hence, there are many more data points than common organisms. For comparison, the top row of the figure shows correlations of a positive control, the α_1_-subunit of the H^+^,K^+^-ATPase with the β_1_-subunit of the H^+^,K^+^-ATPase followed by two negative controls, i.e., PKCα with EGFR followed by hexokinase with the H^+^,K^+^-ATPase α_1_ subunit

Before looking at mirror tree analyses of the α_1_-subunit of the H^+^,K^+^-ATPase with PKC isoforms and members of the Src kinase family, it is important to test the reliability of the mirror tree method using appropriate control systems. As a positive control we have used the α_1_ and β_1_ subunits of the H^+^,K^+^-ATPase. These are in direct physical and chemical contact with one another both within the plasma membrane and when they are in intracellular stores. Therefore, they are an ideal pair of protein polypeptide chains as a positive control of co-evolution. As shown in Fig. 2, the α and β subunits showed a high correlation coefficient of 0.9639, as one would expect for two proteins undergoing co-evolution. Thus, any mirror tree analysis yielding a correlation coefficient of this magnitude can be considered as a strong indication of co-evolution.

For negative controls we used two systems. The first was the α_1_-subunit of the H^+^,K^+^-ATPase and hexokinase. The reason that hexokinase was chosen was that in subsequent analyses, we have studied the correlation of the α_1_-subunit of the H^+^,K^+^-ATPase with protein kinases which could potentially phosphorylate serine or tyrosine residues of the protein. Hexokinase, however, is a kinase which phosphorylates carbohydrates, not proteins. Therefore, one would not expect any co-evolution between hexokinase and the H^+^,K^+^-ATPase. The mirror tree analysis of these two proteins yielded a correlation coefficient of 0.9172 (see Fig. 2). This is an important result, because it indicates that a correlation coefficient of this magnitude can still not be confidently used as a marker for co-evolution. It is not enough to simply look at the value of the correlation coefficient. One must compare the values obtained to others determined for systems where coevolution clearly is or isn’t occurring.

As a second negative control we compared sequences of PKCα and epidermal growth factor receptor (EGFR). EGFR is a protein that is not phosphorylated by serine/threonine kinases such as PKC (Endres et al. 2014) and is, therefore, an ideal test system as a negative control. The analysis yielded a correlation coefficient of 0.7915 (see Fig. 2). Thus, analyses which yield a correlation coefficient of this magnitude cannot be confidently seen as an indicator of coevolution. The control analyses, therefore, indicate that only mirror tree analyses yielding correlation coefficients of > 0.92 can be judged as potential candidates for co-evolution and, thus, potential interaction partners.

### Mirror Tree Analysis of the H^+^,K^+^-ATPase with PKC Isoforms

Mirror tree analyses of the H^+^,K^+^-ATPase α_1_ subunit isoform with each of the isoforms of PKC were conducted. Correlations of the pairwise distances are shown in Fig. 2. The corresponding Pearson correlation coefficients, *r*_AB_; the number of common organisms, *N*; and the number of pairings of the common organisms (i.e., the number of data points in the correlation plots), *n*, are given in Table 1.

**Table 1:** Pearson correlation coefficients, *r*_AB_, for mirror tree analyses of co-evolution of the H^+^,K^+^-ATPase α_1_ subunit and isoforms of PKC. *N* is the number of common organisms, and *n* is the number of pairwise combinations of the common organisms

PKC isoform	r_AB_^*^	N	n
α	0.9240	41	820
β	0.1284	46	1035
γ	0.9132	53	1378
δ	0.7580	67	2211
ε	0.8421	45	990
η	0.9274	55	1485
θ	0.9427	48	1128
ζ	0.9053	36	630
λ	0.6799	51	1275

*In each case, the probability, *p*, of the null hypothesis that there is no significant correlation was < 0.0001

The highest correlation coefficient between the H^+^,K^+^-ATPase and the PKC isoforms was 0.9427 for the θ isoform. The next highest correlation coefficient was 0.9274 for the η isoform. These two isoforms also previously showed the highest correlation coefficients for the α_1_ isoform of the Na^+^,K^+^-ATPase (Blayney et al. 2023). The probability, *p*, of the null hypothesis that the correlation of the H^+^,K^+^-ATPase with the θ isoform is no greater than that with the η isoform was 0.002, or 0.2%. Thus, based on the data, there is strong evidence favouring coevolution of the H^+^,K^+^-ATPase with the PKC θ isoform over all other PKC isoforms. It should be noted, however, that PKCβ undergoes alternative splicing to give two unique isoforms, PKCβ1 and PKCβ2. However, because the Uniprot database, which we used to obtain protein sequences, does not distinguish between these two isoforms, the analysis presented here treated PKCβ as a single isoform. The results obtained for PKCβ should, therefore be treated with some caution.

### Mirror tree Analysis of the H^+^,K^+^-ATPase with Enzymes of the Src Kinase Family

**Fig. 3:**
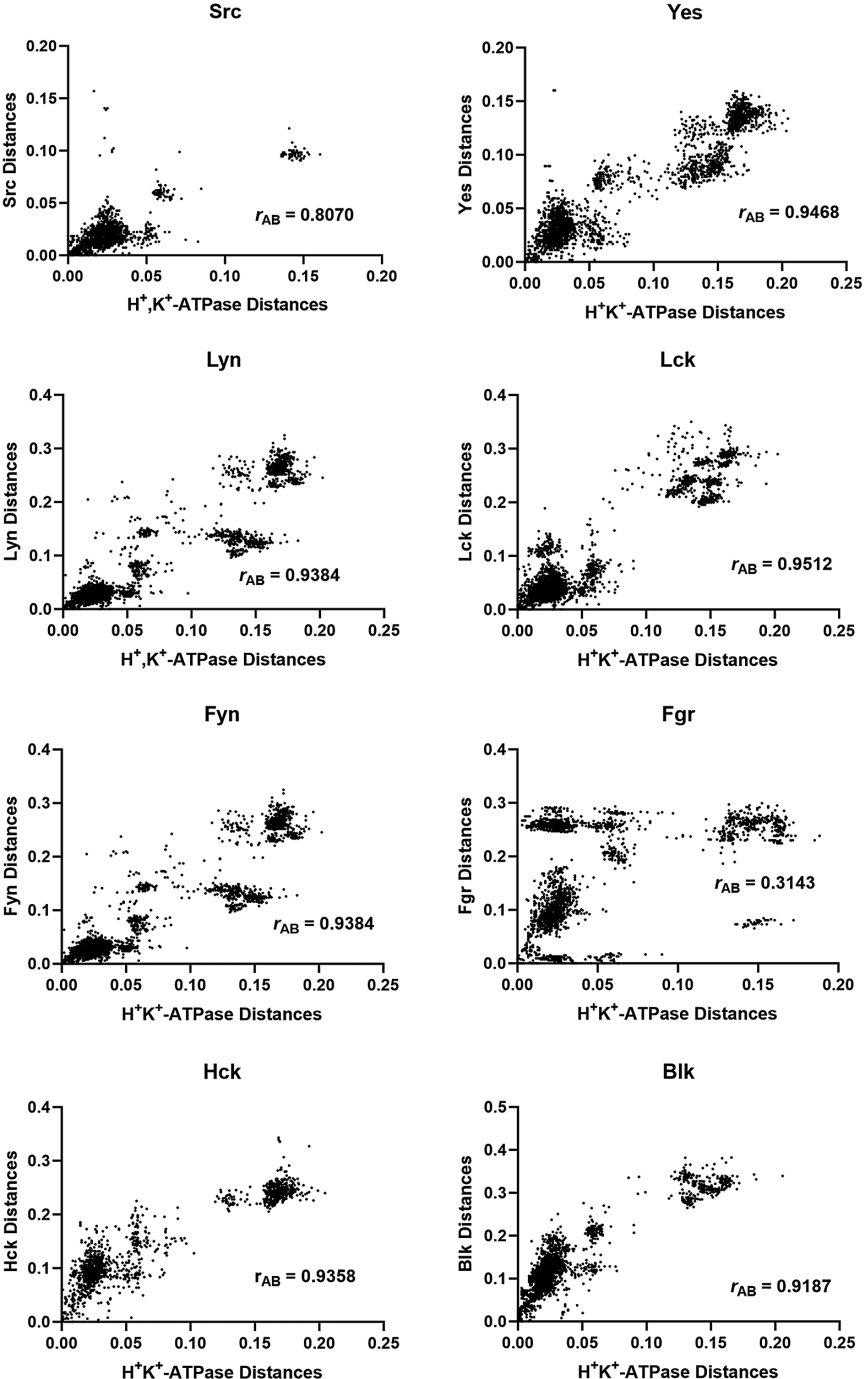
Correlations of evolutionary distances between common organisms that express different members of the Src kinase family of enzymes and the H^+^,K^+^-ATPase α_1_-subunit. Each data point represents a combination of a pair of common organisms for each protein, as explained in the Materials and Methods section

Mirror tree analyses of the H^+^,K^+^-ATPase α_1_ subunit isoform with each member of Src kinase family were also conducted. The correlations of the pairwise distances are shown in Fig. 3. The corresponding Pearson correlation coefficients, *r*_AB_; the number of common organisms, *N*; and the number of pairings of the common organisms, *n*, are given in Table 2.

**Table 2:** Pearson correlation coefficients, *r*_AB_, for mirror tree analyses of co-evolution of the H^+^,K^+^-ATPase α_1_ subunit and members of the Src kinase family. *N* is the number of common organisms, and *n* is the number of pairwise combinations of the common organisms

Src Family Member	r_AB_^*^	N	n
Src	0.8070	83	3403
Yes	0.9468	85	3570
Lyn	0.9384	91	4075
Lck	0.9512	94	4371
Fyn	0.9384	97	4656
Fgr	0.3143	89	3916
Hck	0.9358	61	1830
Blk	0.9187	87	3741

*In each case, the probability, *p*, of the null hypothesis that there is no significant correlation was < 0.0001

The highest correlation coefficient between the H^+^,K^+^-ATPase and the members of the Src kinase family was 0.9512 for Lck. The next highest correlation coefficient was 0.9468 for Yes. The probability, *p*, of the null hypothesis that the correlation of the H^+^,K^+^-ATPase with Lck is no greater than that with Yes was 0.05, or 5%. Thus, based on the data, there is no strong evidence favouring coevolution of the H^+^,K^+^-ATPase with Lck over Yes. The correlation coefficient of Lck was, however, significantly higher than that of all other members of the family (Src, Lyn, Fyn, Hck, Blk and Fgr), with a *p* value of < 0.0001.

## Discussion

The aim of this investigation was to identify the kinases which are most likely to regulate the activity of the gastric H^+^,K^+^-ATPase via an electrostatic switch mechanism. This involves the modulation of an electrostatic interaction between the lysine-rich N-terminus of the α_1_-subunit of the H^+^,K^+^-ATPase and the negatively charged cytoplasmic surface of the surrounding membrane. By introducing the negatively charged phosphate residue onto hydroxyl groups of the N-terminal tail, kinases would reduce the amount of positive charge on the N-terminus, causing its interaction with the membrane to break and be released from the membrane. Three potential sites of phosphorylation on the N-terminus exist: Tyr-7, Tyr-10 and Ser-27. The tyrosine residues are potential targets of kinases of the Src kinase family. Ser-27 is potentially a target for an isoform of PKC.

Sequence alignment of the H^+^,K^+^-ATPase α_1_-subunit (see Fig. 1) shows that Tyr-7 is conserved in all vertebrate species except for cartilaginous fish. Therefore, it is possible that in all vertebrates except cartilaginous fish, the H^+^,K^+^-ATPase might be regulated by a member of the Src kinase family. This family of tyrosine kinases does not have a strict consensus sequence, but it has been reported to prefer neighbouring acidic residues, in particular glutamic acid (E) (Miller 2003). This is here the case, with glutamic acid occupying positions 5 and 8 in most species. If glutamic acid is not in these positions it is usually substituted by the other acidic residue, aspartic acid (D). Therefore, the sequence is consistent with a Src kinase family target.

To determine which Src kinase family member is the most likely interaction partner, the approach we took here was to analyse for evidence of co-evolution. The logic here is that, if two proteins are interacting, then mutations in one are likely to be accompanied by mutations in the other, to maintain an optimal interaction. The bioinformatic method we used was the mirror tree method, which analyses the degree of similarity of two protein’s phylogenetic trees. It was found that, amongst the members of the Src kinase family, the tree of Lck showed the greatest similarity to that of the H^+^,K^+^-ATPase. This is reflected in the correlation coefficient of the plot of pairwise distances of the two trees (see Fig. 3; Table 2). However, at the 5% level of probability, it could not be stated that the correlation with Lck is significantly higher than that of Yes. Therefore, the evidence would suggest that either Lck or Yes regulate the gastric H^+^,K^+^-ATPase by phosphorylation of Tyr-7.

Tyr-10 is also relatively close to glutamic acid residues, in positions 8 and 13 in most species, consistent with a target sequence for a Src kinase family member. However, Tyr-10 is less conserved than Tyr-7. In mammals it is often substituted by a histidine residue (H) and in all bony fish by phenylalanine (F). Therefore, if Tyr-10 is a regulatory site, then it can only function in a more specialized role in some vertebrate species.

Ser-27 is a potential target for a PKC isoform. Mirror tree analysis shows that the θ isoform would be the most likely interaction partner (see Fig. 2; Table 1). Ser-27 is preceded by the positively charged basic residue lysine (K) in position 25 in many species and it is followed by multiple lysines in all species where it is present. This is consistent with a consensus sequence for a PKC (Rust and Thompson 2011). However, Ser-27 is not completely conserved across any class of vertebrates, not even placental mammals. Therefore, it cannot be involved in a universal mechanism of H^+^,K^+^-ATPase regulation in vertebrates.

In summary, based on the bioinformatic data available it would seem the most likely enzyme candidates involved in acute regulation of the H^+^,K^+^-ATPase are Lck and Yes through phosphorylation of Tyr-7. Lck stands for “lymphocyte-specific protein tyrosine kinase”. Thus, its major tissue of expression is in the lymph nodes. However, according to expression data available on the National Library of Medicine’s National Center for Biotechnology Information (NCBI) gene database (https://www.ncbi.nlm.nih.gov/gene/3932#gene-expression), in humans there is also a lower level of expression in the stomach. Yes is more broadly expressed, with a significant expression in the stomach (https://www.ncbi.nlm.nih.gov/gene/7525#gene-expression). Its name appears to be derived from the fact that it is the cellular homologue of the Yamaguchi sarcoma virus oncogene. As far as we are aware, the bioinformatic data presented here is the first indication that acid secretion in the stomach could be under acute regulation in vivo via protein kinases. Nevertheless, the coevolution correlations observed cannot be taken as proof of kinase regulation. Experimental studies must be performed. But considering the number of different kinase isoforms that exist, the results presented here will help to focus future experimental work.

## Data Availability

Data is provided within the manuscript.
